# A Handbook of Obstetric Nursing; Comprising the Course of Instruction in Obstetric Nursing Given to the Pupils of the Training School for Nurses Connected with the Woman’s Hospital of Philadelphia

**Published:** 1899-06

**Authors:** 


A Handbook of Obstetric Nursing ; Comprising the Course of Instruction in Obstetric Nursing given to the Pupils of the Training School for Nurses connected with the Woman's Hospital of Philadelphia. By Anna M. Fullerton. M. D., Demonstrator of Obstetrics in the Woman's Medical College of Pennsylvania ; Physician-in-Charge and Obstetrician and Gynecologist to the Woman's Hospital of Philadelphia, and Superintendent of the Nurses Training School of the Woman's Hospital of Philadelphia. Forty illustrations ; 12mo. Fourth edition, revised. Philadelphia : P. Blakiston's Son & Co. 1899.

The fifth edition of Dr. Fullerton's handbook on obstetric nursing has been revised and brought up-to-date. The manual comprises the course of instruction given the pupils of the training-school for nurses, connected with the Woman's Hospital of Philadelphia. It will be found valuable, both as a text-book on obstetric nursing in hospital training-schools, and as a guide to mothers and others who wish to inform themselves on the care of the pregnant and parturient woman. M. J. F.

